# Effectiveness of robust optimization in volumetric modulated arc therapy using 6 and 10 MV flattening filter-free beam therapy planning for lung stereotactic body radiation therapy with a breath-hold technique

**DOI:** 10.1093/jrr/rraa026

**Published:** 2020-05-05

**Authors:** Hideharu Miura, Shuichi Ozawa, Yoshiko Doi, Minoru Nakao, Katsumaro Kubo, Masahiko Kenjo, Yasushi Nagata

**Affiliations:** 1 Hiroshima High-Precision Radiotherapy Cancer Center; 2 Department of Radiation Oncology, Institute of Biomedical & Health Sciences, Hiroshima University

**Keywords:** robust optimization, lung, VMAT, dose uncertainty, perturbed dose

## Abstract

We investigated the feasibility of a robust optimization with 6 MV X-ray (6X) and 10 MV X-ray (10X) flattening filter-free (FFF) beams in a volumetric modulated arc therapy (VMAT) plan for lung stereotactic body radiation therapy (SBRT) using a breath-holding technique. Ten lung cancer patients were selected. Four VMAT plans were generated for each patient; namely, an optimized plan based on the planning target volume (PTV) margin and a second plan based on a robust optimization of the internal target volume (ITV) with setup uncertainties, each for the 6X- and 10X-FFF beams. Both optimized plans were normalized by the percentage of the prescription dose covering 95% of the target volume (D_95%_) to the PTV (1050 cGy × 4 fractions). All optimized plans were evaluated using perturbed doses by specifying user-defined shifted values from the isocentre. The average perturbed D_99%_ doses to the ITV, compared to the nominal plan, decreased by 369.1 (6X-FFF) and 301.0 cGy (10X-FFF) for the PTV-based optimized plan, and 346.0 (6X-FFF) and 271.6 cGy (10X-FFF) for the robust optimized plan, respectively. The standard deviation of the D_99%_ dose to the ITV were 163.6 (6X-FFF) and 158.9 cGy (10X-FFF) for the PTV-based plan, and 138.9 (6X-FFF) and 128.5 cGy (10X-FFF) for the robust optimized plan, respectively. Robust optimized plans with 10X-FFF beams is a feasible method to achieve dose certainty for the ITV for lung SBRT using a breath-holding technique.

## Introduction

Stereotactic body radiation therapy (SBRT) has played an increasingly important role in the non-surgical treatment of stage I non-small-cell lung cancer. Multiple institutions have achieved a high local control rate [1, 2] with limited toxicity [3, 4]. The lungs are among the most radiation-sensitive organs, with two different manifestations of radiation damage, of both early and late occurrences. Management of respiratory motion is an important issue for lung SBRT. The management of respiratory motion, along with a comprehensive review of different methods, can be found in a report by Task Group 76 of the American Association of Physicists in Medicine [5]. Respiratory management techniques utilize a specific technology that requires longer treatment times for the delivery of a precise treatment. The breath-holding technique is a non-invasive and cost-effective approach for minimising tumour motion, resulting in the delivery of high doses to the planning target volume (PTV), while maximally sparing the dose to the organ at risk (OAR). Volumetric modulated arc therapy (VMAT) has been used effectively in combination with flattening filter-free (FFF) beams with higher dose rates to reduce the SBRT delivery to < 3 min/fraction without compromising the plan quality or dosimetric accuracy [6]. Although the techniques applied to lung SBRT and their clinical evidence have been dramatically strengthened [7–9], several problems regarding lung cancer treatment planning remain unsolved.

For lung SBRT, a significant portion of the PTV contains low-density lung tissue. During plan optimization, the PTV requires targeting because the gross tumour volume (GTV) can exist anywhere inside the PTV. For VMAT, the plan optimization increases the fluence to compensate for the lack of electronic equilibrium in a low-density region, resulting in the delivery of higher doses to the GTV and lung tissue at the edges of the GTV. Overriding the electron densities of the internal target volume (ITV) or PTV is a useful method for reducing the extra fluence required to compensate for the dose at the edge of the PTV [10]. Another problem of lung SBRT is related to the dose prescription. The PTV is designed to ensure that the tumour receives an adequate absorbed dose. A PTV D_95%_ prescription has been widely used in VMAT to account for position uncertainties relative to the target volume during treatment delivery. In the lung region, the PTV D_95%_ cannot be used to predict the minimum dose to the GTV because the PTV margin mostly consists of a low-density region. The GTV dose should be evaluated because the PTV prescription cannot be used to predict the dose to the GTV. Prescriptions based on the GTV or ITV for lung SBRT have been proposed to solve this issue [11–13]. These prescriptions are required to simulate the dose for patient position uncertainties because the GTV may be overexposed when it moves into regions with increased photon fluence. To solve this issue, robust optimization algorithms are required to account for patient position uncertainties [14, 15]. Liang et al. reported that an ITV-based optimized plan using robust optimization can be applied to reduce the dose to the normal lung tissue while maintaining the dose coverage when a setup error occurs [14]. Their experimental plans were generated using a 6 mega-voltage (MV) X-ray (6X) with a flattening filter (FF). Some reports have indicated the differences between FF and FFF beams for lung tumours [16, 17]. Hrbacek et al. presented a planning comparison of 6X-FF VMAT, 6X-FFF VMAT, and 10 MV X-ray (10X) FFF VMAT SBRT for lung tumours, focusing on a limited number of dosimetric variables. They concluded that the 10X-FFF beam improves the treatment efficiency and provides lower skin and peripheral doses. The aforementioned studies only explored VMAT techniques with regard to a PTV-based optimization method. In addition, few papers have been published on FFF beams using the robust optimization method for lung SBRT treatment. The fluence of an FFF beam may influence the dosimetric outcome for lung cancer, because a secondary build-up and lateral disequilibrium may affect target coverage. Verification of the accuracy of dose delivery before the introduction of a new technique into clinical practice is very important to ensure that the treatment is correct. The modulation complexity score for VMAT (MCSv) is used to assess the variability of leaf positions and aperture areas between segments, which are indexed to the complexity of the treatment plan [18, 19].

This study aims to assess the differences between 6X- and 10X-FFF beams using PTV-based and robust optimizations for lung cancer patients using a breath-holding technique. Lung VMAT SBRT plans incorporating PTV-based and robust optimized plans using 6X- and 10X-FFF beams were generated and evaluated for perturbation doses to the ITV and lung. The plan complexity of both optimized plans was investigated.

## Materials and Methods

### Treatment planning

This study was approved by the Institutional Review Board of Hiroshima University (E-1223). Ten patients with stage I non-small-cell lung cancer, treated using a breath-holding VMAT SBRT, were chosen for the analysis. The patient characteristics are provided in Table 1. Breath-holding techniques can reduce the ITV and PTV for both treatment planning and delivery. The reproducibility of the tumour position was confirmed within 5 mm using an X-ray fluoroscopy simulator (VersiFlex VISTA, Hitachi Medical Co., Kashiwa, Japan). Abches (Apex Medical Inc., Tokyo, Japan) was used to monitor the self-control of the respiratory motion, as well as the tumour displacement. While applying a breath holding technique, all patients underwent a computed tomography (CT) scan (Optima CT 580 W; GE Healthcare, Milwaukee, WI, USA) conducted in a supine position with their arms above their head using an arm rest. The CT images had a slice thickness of 1.25 mm with a gantry rotation time of 0.5 s. Multiple CT scans were performed to evaluate the inter-fraction breath-hold reproducibility of the tumour position at the expiratory breath-hold. The first CT scan at the expiratory breath-hold was used for treatment planning. The remaining three CT scans were acquired successively without repositioning to simulate inter-fractional expiratory breath-hold reproducibility. All VMAT plans were contoured, optimized, and calculated based on a breath-holding CT image dataset used in the RayStation treatment planning system (TPS) version 6.2.0 (RaySearch Medical Laboratories AB, Stockholm, Sweden) commissioned through a TrueBeam STx (Varian Medical Systems, Palo Alto, CA) linear accelerator. The GTV was contoured in the CT pulmonary window by a radiation oncologist. No additional margin was added to the GTV for generation of the clinical target volume (CTV). The ITV was created with a 3 mm expansion from the CTV to account for uncertainties in the breath holding of the patient. Although inter-fraction breath-hold reproducibility for all case was less than 3 mm on multiple CT scans, a fixed margin of 3 mm was applied rather than individually customized margins. The PTV was further defined by adding an isotropic margin of 5 mm to the ITV to account for uncertainties and mechanical inaccuracies in the setup. The CTV was 5.5 ± 3.7 cm^3^ (0.8–15.3 cm^3^), ITV was 12.0 ± 6.8 cm^3^ (3.0–29.2 cm^3^), and PTV was 31.6 ± 10.9 cm^3^ (18.9–57.4 cm^3^). A ‘PTV + 3 mm’ ring was created to conform the dose to the PTV, and a ‘PTV + 8 mm’ ring was used to reduce the dose to healthy tissues. The lung, trachea, main bronchus, esophagus, spinal cord, and skin were contoured as OARs and a single constraint limiting the maximum dose to these rings and OAR structures was applied.

**Table 1:** Patient characteristics

Patient	Male/female	Age (y)	Tumour location	CTV (cm^3^)	ITV (cm^3^)	PTV (cm^3^)
1	M	76	LUL	6.8	14.7	35.3
2	M	83	RUL	4.2	11.1	30.5
3	M	94	LLL	7.1	15.1	36.8
4	M	83	RML	2.8	7.6	22.0
5	M	70	LUL	15.3	29.2	57.4
6	M	61	RUL	5.3	12.8	32.8
7	F	70	RLL	0.8	3.0	18.9
8	F	86	RML	4.5	6.2	19.2
9	F	74	LUL	3.4	9.1	25.4
10	M	81	RLL	4.5	11.6	37.8
Mean	-	78	-	5.5	12.0	31.6

M; male, F; female, CTV; clinical target volume, ITV; internal target volume, PTV; planning target volume, LUL; left upper lobe, LLL; left lower lobe, RUL; right upper lobe, RML; right middle lobe, and RLL; right lower lobe.

The RayStation system offers a minimax optimization, in which the optimization functions that are selected as robust are considered under the worst-case scenario [20]. The interfractional patient-setup uncertainties are considered to be random; they are incorporated by shifting the isocentre of the patient in the anterior-posterior (A-P), superior-inferior (S-I), and right-left (R-L) directions by the same margin as those used for defining the PTV. This yields six dose distributions delineated by

(1) \begin{equation*} \underset{x\in X}{\min}\ \underset{s\in S}{\max}\sum_{i=1}^n{W}_i{f}_i\left(d\left(x;s\right)\right) \end{equation*}

where *W* is the weight, *f* is the function, *X* is the set of feasible variables, *d(x;s)* is the dose distribution as a function of the variables *x* and scenario *s*, and *n* is the number of objective functions. Optimizing with robust objectives under several scenarios is an iterative process, potentially requiring more iterations to obtain an equivalent objective function value.

The isocentre was placed at the centre of the tumour. For each patient, single-arc VMAT plans were prepared using PTV-based optimizations of nominal energy 6X-FFF PTV-based plan and 10X-FFF (PTV-based plans) beams with a 5 mm uniform PTV margin from the ITV, and robust optimizations of nominal energy 6X-FFF and 10X-FFF(robust plans) beams with 5 mm setup uncertainties, resulting in a total of four plans. For each beam, the maximum available dose rate was used; 1,400 and 2,400 monitor units per minute (MU/min) for the 6X- and 10X-FFF beams, respectively. The arc angles were chosen to cover the target while sparing the contralateral lung and other critical OARs. The robust optimized plans were generated using the same arc angles used in the PTV-based optimized plans. The collimator angle was fixed at 10°. For the dose calculations, a collapsed cone convolution algorithm was applied with a grid resolution of 2.0 mm. The dose prescription was 42 Gy with 10.5 Gy per fraction. The Japanese standard dose of 48 Gy in four fractions was prescribed at the isocentre, equal to 42 Gy in four fractions at the D_95%_ of the PTV [21]. The PTV was not necessary for the robust optimized plan, but was used for dose normalization. The dose was prescribed inhomogeneously to the PTV with near-maximum dose (D_2%_) and ranged from 125% to 130%. The dose constraints of the OAR, including the lung, trachea, main bronchus, esophagus, heart, spinal cord, and skin under our clinical protocol are provided in Table 2.

**Table 2:** Dose and volume constraints for organs at risk

Organ	Dose	Volume
Lung	V_15Gy_	≦25%
	V_20Gy_	≦20%
	Mean dose	≦18 Gy
Trachea	V_40Gy_	≦10 cm^3^
Main bronchus	V_40Gy_	≦10 cm^3^
Esophagus	V_40Gy_	≦1 cm^3^
Heart	V_30Gy_	≦15 cm^3^
Spinal cord	25 Gy	Maximum
Skin	40 Gy	Maximum

Vx, percentage of volume receiving x Gy of the prescribed dose.

For the nominal plan (no setup uncertainty), the values of the doses received by 99%, 98%, 50%, and 2% of the volume (D_99%_, D_98%_, D_50%_, and D_2%_, respectively) were measured, using the dose volume histogram (DVH). The homogeneity index (HI) of the treatment was calculated using the following formula [22]:

(2) \begin{equation*} \mathrm{HI}=\frac{D_{2\%}-{D}_{98\%}}{D_{50\%}} \end{equation*}

The conformity index (CI) was calculated using the following formula [23]:

(3) \begin{equation*} CI=\frac{TV_{PIV}^2}{\left( TV\times{V}_{RI}\right)}, \end{equation*}

where TV is the target volume, TV_PIV_ is the target volume covered by the prescription isodose, and V_RI_ is the total volume covered by the prescription isodose. The percentages of the total lung minus the GTV receiving 20 Gy (V_20Gy_), 5 Gy (V_5Gy_), and the mean dose were evaluated. The number of MUs was recorded. The actual delivery time was recorded, excluding any time required for additional imaging and setup.

### Perturbed dose evaluation

The robustness of each plan was evaluated by calculating the perturbed doses from 5 mm spatial shifts under 14 scenarios (6 with 5 mm shifts in the A-P, S-I, and R-L directions and 8 with 5 mm shifts in the diagonal directions). For the ITV, the evaluation metrics included the perturbed doses of D_99%_, D_98%_, D_50%_, and D_2%_. For the lung, the V_20Gy_, V_5Gy_, and mean dose were evaluated as well as the HI and CI.

### Plan complexity

We used the MCSv to assess the plan complexities for the PTV-based and robust optimizations [18]. The MCSv was calculated using the variability of the leaf positions and the aperture area between control points, as originally proposed by McNiven et al. [19]. The MCSv is a single metric ranging from 0 to 1.0 such that an open rectangular field with no complexity has an MCS of 1.0. The value of MCSv decreases with increasing multileaf collimator (MLC) sequence modulation. In our study, the MCSv was calculated using the Digital Information and Communication in Medicine Radiation Therapy (DICOM-RT) files and Microsoft Visual C#.

### Data analysis

The data were analysed using Wilcoxon signed-rank tests with the statistical significance set at *p* < 0.05, applying R version 3.5.1 (www.r-project.org).

## Results

### Treatment plans

**Fig. 1.**
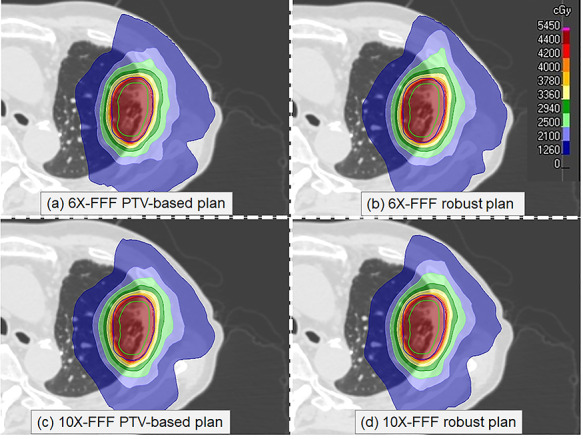
Comparison of nominal dose distributions created by different planning techniques for a patient: (a) PTV-based and (b) robust optimizations using 6X-FFF beams; and (c) PTV-based and (d) robust optimizations using 10X-FFF beams. The ITV and PTV are shown in green and blue, respectively.

**Fig. 2.**
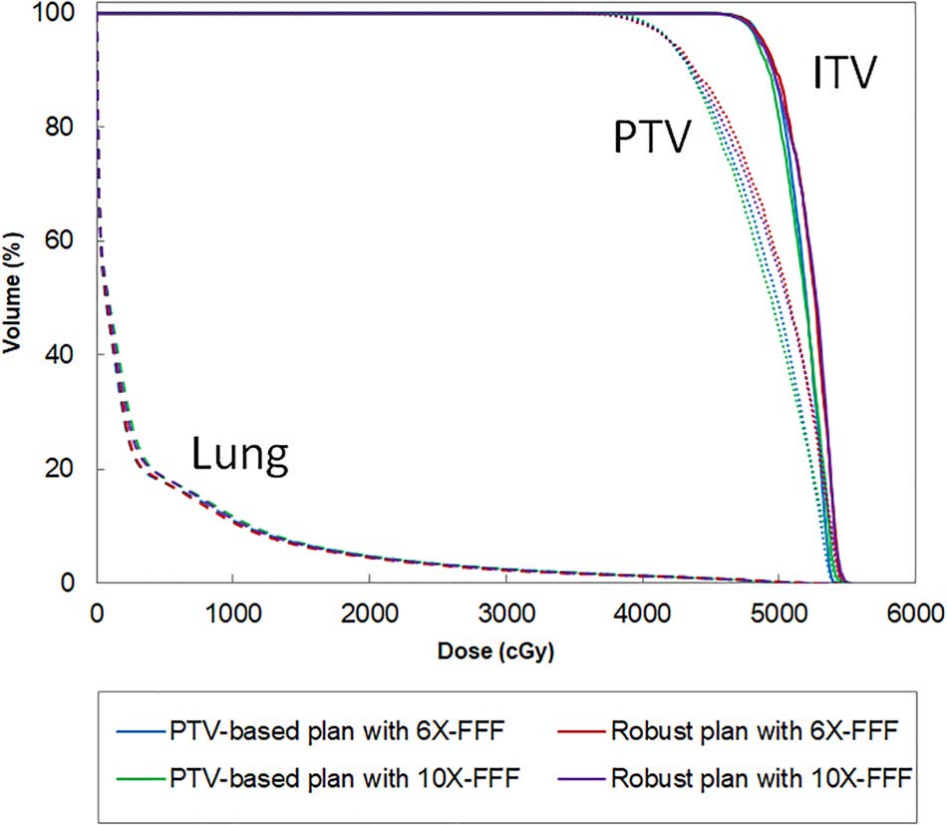
Comparison of the nominal DVHs (solid line: ITV, dot line: PTV and dashed line: lung) created using different planning techniques for the same patient as in Fig. 1: PTV-based optimization using 6X-FFF (blue), robust optimization using 6X-FFF (red), PTV-based optimization using 10X-FFF (green), and robust optimization using 10X-FFF (purple) beams.

The dose distributions and DVHs of the ITV and lung doses for the four plans are shown for patient No. 5 in Figs. 1 and 2, respectively. The corresponding quantitative evaluations from the DVH analysis of all patients are listed in Table 3. The dose constraint to the OARs was met by all techniques.

**Table 3:** Doses to the ITV and lung, HI, CI, MU, and delivery times using PTV-based and robust optimized plans with 6X- and 10X-FFF beams

	PTV-based plan 6X-FFF	Robust plan 6X-FFF	PTV-based plan 10X-FFF	Robust plan 10X-FFF	*p*
ITV: D_99%_ (cGy)	4693.6 ± 84.7 (4587.0–4850.0)	4730.7 ± 78.1 (4609.0–4867.0)	4644.1 ± 110.3 (4539.0–4901.0)	4633.0 ± 140.2 (4313.0–4871.0)	d
ITV: D_98%_ (cGy)	4741.9 ± 84.4 (4599.0–4874.0)	4776.7 ± 81.6 (4627.0–4902.0)	4690.5 ± 117.1 (4572.0–4970.0)	4687.2 ± 142.1 (4391.0–4942.0)	d
ITV: D_50%_ (cGy)	5120.6 ± 61.2 (5012.0–5215.0)	5161.2 ± 58.0 (5067.0–5262.0)	5118.7 ± 123.2 (4934.0–5304.0)	5162.7 ± 114.9 (4996.0–5306.0)	b, c
ITV: D_2%_ (cGy)	5367.5 ± 44.4 (5300.0–5454.0)	5390.2 ± 45.2 (5330.0–5456.0)	5411.0 ± 52.8 (5283.0–5460.0)	5391.7 ± 44.9 (5300.0–5460.0)	c
ITV: HI	0.122 ± 0.020 (0.098–0.159)	0.119 ± 0.024 (0.083–0.159)	0.141 ± 0.020 (0.093–0.163)	0.151 ± 0.031 (0.083–0.209)	a,d
CI	1.04 ± 0.06 (0.88–1.10)	1.05 ± 0.07 (0.84–1.14)	1.05 ± 0.09 (0.81–1.13)	1.06 ± 0.08 (0.84–1.13)	-
Lung: mean dose (cGy)	316.8 ± 57.1 (216.0–394.0)	306.6 ± 59.5 (206.0–393.0)	352.3 ± 65.3 (228.0–435.0)	336.1 ± 72.9 (221.0–448.0)	a,b,d
Lung: V_20Gy_ (%)	4.1 ± 1.0 (2.4–5.5)	3.8 ± 1.0 (2.2–5.3)	4.6 ± 1.3 (2.4–6.7)	4.4 ± 1.4 (2.3–6.9)	a,b,c,d
Lung: V_5Gy_ (%)	15.5 ± 2.6 (11.6–18.8)	14.2 ± 3.1 (10.0–19.3)	17.1 ± 3.0 (11.8–21.2)	16.3 ± 3.7 (11.2–22.5)	a,b,d
MU	1787 ± 91 (1607–1895)	1815 ± 97 (1641–1949)	1762 ± 103 (1590–1897)	1790 ± 79 (1679–1891)	b
Delivery time (sec)	77 ± 3.8 (70–81)	78 ± 4.2 (70–85)	49 ± 2.1 (44–52)	49 ± 3.3 (42–53)	a,d

Group averages; the ranges are shown in parentheses (n = 10). ^*^The Wilcoxon signed-rank test resulted in a statistically significant difference (*p* < 0.05). a, PTV-based 6X-FFF vs. 10X-FFF; b, 6X-FFF PTV-based vs. robust; c, 10X-FFF PTV based vs. robust; d, robust 6X-FFF vs. 10X-FFF; FFF, flattening filter free; HI, homogeneity index; CI, conformity index; MU, monitor unit; D_x%_, dose received by x% of the volume of interest; Vx, percentage of volume receiving x Gy of the prescribed dose.

#### PTV-based optimized plan comparisons

Only minor differences were observed in the achieved dose distributions and corresponding DVHs when comparing the two plans (6X-FFF PTV-based and 10X-FFF PTV-based) for the same patient (Fig. 1 (a) and (c)). ITV doses were no significant differences between both 6X- and 10X-FFF beams. There was a significant difference between the HIs (p = 0.006). The CIs were similar for the 6X- and 10X-FFF beams (p = 0.625). Compared to the 10X-FFF beams, the mean dose, V_20Gy_, and V_5Gy_ for the lungs with the 6X-FFF beams were on average decreased by 11.2% (p = 0.004), 13.0% (p = 0.011), and 10.0% (p = 0.027), respectively. The MUs obtained by the 6X- and 10X-FFF beams were similar (p = 0.160). The average ± standard deviation delivery times for the 6X- and 10X-FFF beams were 77.0 ± 3.8 s and 49.0 ± 2.1 s, respectively (p = 0.002).

#### Robust optimized plan comparisons

Only minor differences were observed for the achieved dose distributions of the two plans (6X- and 10X-FFF-robust plans) for the same patient (Fig. 1 (b) and (d)). For the robust optimized plans, the D_99%_ and D_98%_ doses to the ITVs exhibited statistically significant differences for the 6X- and 10X-FFF beams (D_99%_: p = 0.010, D_98%_: p = 0.040).There was also a significant difference between the HIs (p = 0.039). The CIs were similar for the 6X- and 10X-FFF beams (p = 0.437). Compared to the 6X-FFF beams, the mean dose, V_20Gy_, and V_5Gy_ to the lung for the 10X-FFF beam were increased by 9.6% (p = 0.002), 15.0% (p = 0.004), and 14.6% (p = 0.004) on average, respectively. Differences in the MUs were not found to be statistically significant for either of the FFF beams (p = 0.106). The average ± standard deviation delivery times for the 6X- and 10X-FFF beams were 78.0 ± 4.2 and 49.0 ± 3.3 s, respectively (p = 0.002).

#### Comparison between PTV-based and robust optimized plans

For the 6X-FFF beams, the ITV doses showed no statistically significant differences between the PTV-based and robust optimized plans. There was no significant difference between the HIs. The CI were similar for PTV-based and robust optimized plans (p = 0.625). Compared to the PTV-based optimized plans with 6X-FFF beam, the mean dose, V20Gy, and V5Gy to the lung were with the robust optimized plans on average reduced by 3.2% (p = 0.002), 7.4% (p = 0.002), and 8.5% (p = 0.014), respectively. The average of MUs for robust optimized plans was slightly higher than that PTV-based optimized plans. The average ± standard deviation delivery times for the 6X-FFF PTV-based and 6X-FFF robust optimized plans were 77.0 ± 3.8 s and 78.0 ± 4.2 s, respectively (p = 0.002). The 10X-FFF robust optimized plans also indicated a similar tendency for CI, lung dose, MUs, and delivery time.

### Perturbed dose evaluation

**Fig. 3.**
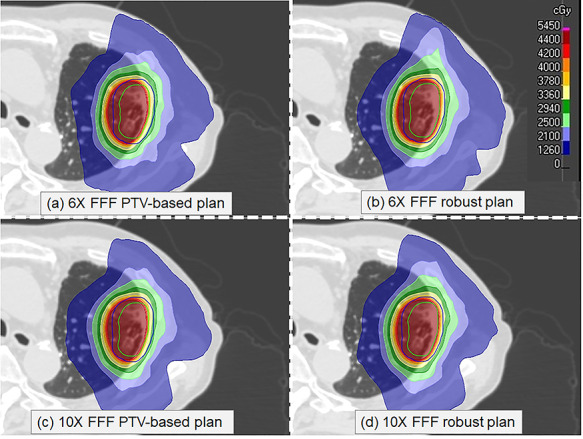
Comparison of the perturbed dose distributions with a 5 mm right-direction isocentre shift created by different planning techniques for the same patient as in Fig. 1: (a) PTV-based optimization using 6X-FFF, (b) robust optimization using 6X-FFF, (c) PTV-based optimization using 10X-FFF, and (d) robust optimization using 10X-FFF beams. The ITV and PTV are shown in green and blue, respectively.

**Fig. 4.**
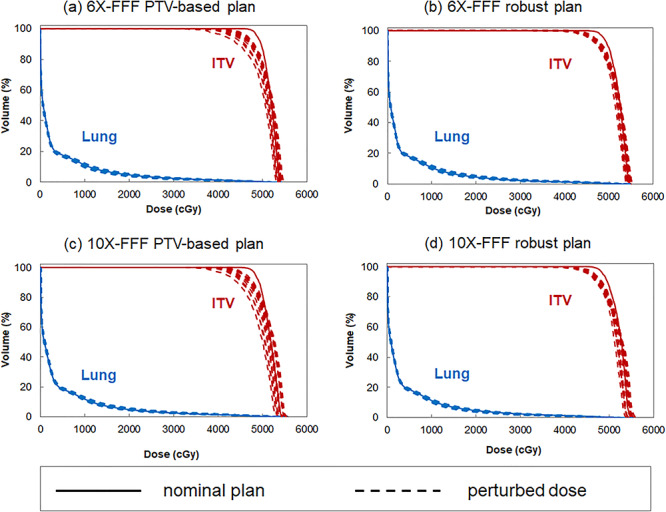
Plan robustness evaluations, shown as DVHs for the same patient as in Fig. 1: (a) PTV-based optimization using 6X-FFF, (b) robust optimization using 6X-FFF, (c) PTV-based optimization using 10X-FFF, and (d) robust optimization using 10X-FFF beams (solid line: nominal plan and dashed line: perturbed dose evaluation).

The perturbed dose distributions and DVHs of the ITV and lung doses, with a 5 mm rigidly right-direction shifted isocentre for the four plans applied to patient No. 5, are shown in Figs. 3 and 4, respectively. Table 4 compares the ITV, HI, CI, and lung doses obtained from the rigidly 5 mm isocentre shifted plan, for the PTV-based and robust optimized plans with an overall perturbation.

**Table 4:** Doses for the ITV, lung, HI, and CI for each plan at offset locations using PTV-based and robust optimized plans with 6X- and 10X-FFF beams

	PTV-based plan 6X-FFF	robust plan 6X-FFF	PTV-based plan 10X-FFF	robust plan 10X-FFF	*p*
ITV: D_99%_ (cGy)	4324.5 ± 163.6 (3721.0–4745.0)	4384.7 ± 138.9 (4130.0–4721.0)	4343.1 ± 158.9 (3753.0–4748.0)	4361.5 ± 128.5 (4145.0–4765.0)	a,b,c,d
ITV: D_98%_ (cGy)	4427.2 ± 139.3 (3807.0–4748.0)	4491.3 ± 116.7 (4225.0–4771.0)	4427.9 ± 141.8 (3867.0–4785.0)	4456.3 ± 116.3 (4202.0–4806.0)	b, c
ITV: D_50%_ (cGy)	5089.0 ± 69.6 (4947.0–5240.0)	5130.3 ± 68.0 (4964.0–5272.0)	5086.4 ± 129.5 (4884.0–5361.0)	5132.9 ± 120.3 (4927.0–5362.0)	b,c
ITV: D_2%_ (cGy)	5364.7 ± 64.1 (5160.0–5483.0)	5388.2 ± 67.7 (5183.0–5511.0)	5408.1 ± 82.3 (5221.0–5629.0)	5455.4 ± 76.2 (5249.0–5608.0)	a,b,c,d
ITV: HI	0.174 ± 0.037 (0.064–0.294)	0.166 ± 0.036 (0.064–0.227)	0.193 ± 0.030 (0.104–0.281)	0.195 ± 0.028 (0.087–0.250)	a,b,d
CI	0.85 ± 0.04 (0.74–0.94)	0.85 ± 0.04 (0.65–0.93)	0.83 ± 0.05 (0.74–0.97)	0.83 ± 0.04 (0.74–0.93)	a,b,d
Lung: mean dose (cGy)	315.8 ± 58.4 (200.0–409.0)	306.3 ± 61.2 (192.0–408.0)	340.3 ± 68.3 (213.0–451.0)	336.1 ± 72.0 (207.0–465.0)	a,b,c,d
Lung:V_20Gy_ (%)	4.1 ± 1.0 (2.0–5.8)	3.8 ± 1.0 (1.2–5.5)	4.4 ± 1.4 (2.0–7.0)	4.4 ± 1.4 (2.0–7.3)	a,b,c,d
Lung:V_5Gy_ (%)	15.5 ± 2.6 (11.1–19.6)	15.0 ± 3.0 (10.3–20.2)	16.3 ± 3.2 (11.0–22.1)	16.3 ± 3.7 (10.5–23.5)	a,b,d

Group averages; the ranges are shown in parentheses (n = 10). ^*^The Wilcoxon signed-rank test resulted in a statistically significant difference (*p* < 0.05). a, PTV-based 6X-FFF vs. 10X-FFF; b, 6X-FFF PTV-based vs. robust; c, 10X-FFF PTV based vs. robust; d, robust 6X-FFF vs. 10X-FFF; FFF, flattening filter free; HI, homogeneity index; CI, conformity index; D_x%_, dose received by x% of the volume of interest; Vx, percentage of volume receiving x Gy of the prescribed dose.

#### PTV-based optimized plan comparisons

The D_99%_ dose to the ITV using the 10X-FFF beam was 18.6 cGy higher on average than that of the 6X-FFF beam (p = 0.067). The ITV perturbed doses of D_99%_ for the 6X- and 10X-FFF beams were 124.5 cGy and 143.1 cGy higher on average than the prescription dose, respectively. The standard deviation of the D_99%_ dose to the ITV for the 6X- and 10X-FFF beams were 163.6 and 158.9 cGy, respectively. The mean dose, V_20Gy_, and V_5Gy_ to the lung using the 10X-FFF beam increased on average by 7.8% (p < 0.001), 8.4% (p < 0.001), and 5.4% (p < 0.001), compared with those of the 6X-FFF beam, respectively.

#### Robust optimized plan comparisons

The D_99%_ dose to the ITV using the 10X-FFF beam was 23.2 cGy lower on average than that of the 6X-FFF beam (p < 0.001). The D_99%_ dose to the ITV using the 6X- and 10X-FFF beams were on average 184.7 cGy and 161.5 cGy higher than the prescription dose, respectively. The standard deviations of D_99%_ to the ITV for the 6X- and 10X-FFF beams were 138.9 and 128.5 cGy, respectively. The mean dose, V_20Gy_, and V_5Gy_ to the lung using a 10X-FFF beam increased by 9.7% (p < 0.001), 15.7% (p < 0.001), and 8.7% (p < 0.001) on average, respectively, compared with those of the 6X-FFF beam.

#### Comparison between PTV-based and robust plans

The ITV perturbed doses of D_99%_ for the 6X-FFF PTV-based and robust optimized plans were on average higher 124.5 cGy vs 143.1 cGy higher than the prescription dose, respectively, and 184.7 cGy vs 161.5 cGy for the 10X-FFF PTV-based and robust optimized plans, respectively. The robust optimized plans provide an improvement in the uncertainty of the ITV doses, as compared with the PTV-based plans. The standard deviation of the D_99%_ dose to the ITV for the 6X- and 10X-FFF beams decreased from 163.6 to 138.9 cGy and 158.9 to 128.5 cGy, respectively. The mean dose, V_20Gy_, and V_5Gy_ to the lung using a robust optimized plans with 6X-FFF beam reduced by 3.0% (p < 0.001), 7.7% (p < 0.001), and 3.3% (p < 0.001) on average, respectively, compared with those of the PTV based optimized plans. The mean dose, V_20Gy_, and V_5Gy_ to the lung using a robust optimized plans with 10X-FFF beam reduced by 4.7% (p < 0.001), 0.2% (p < 0.001), and 1.5% (p < 0.001) on average, respectively, compared with those of the PTV based optimized plans.

### Comparison between the nominal plan and perturbed dose evaluation

**Fig. 5.**
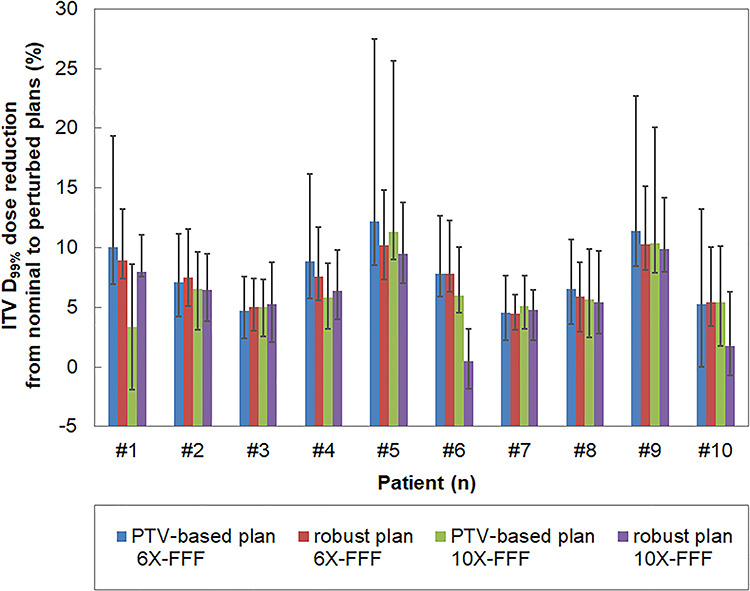
Reduction rate of the ITV dose from the nominal to perturbed plans of the PTV-based optimization using 6X-FFF, robust optimization using 6X-FFF, PTV-based optimization using 10X-FFF, and robust optimization using 10X-FFF beams. The bars indicate the average values: the upper and lower whiskers indicate the maximum and minimum values, respectively.

Figure 5 shows the D_99%_ dose reduction to the ITV, from the nominal plan to the perturbed evaluation, for all patients. The average (and range of) perturbed D_99%_ doses to the ITV compared to the nominal plan decreased by 369.1 cGy (1.0–1022.0 cGy, 6X-FFF beam) and 301.0 cGy (−87.0–964.0 cGy, 10X-FFF beam) for the PTV-based plan, and 346.0 cGy (136.0–629.0 cGy, 6X-FFF beam) and 271.6 cGy (−79.0–591.0 cGy, 10X-FFF beam) for the robust optimized plan. The robust optimized plans achieved higher ITV dose certainties compared with the PTV-based optimized plans.

### Plan complexity

The average ± standard deviation (and range of) MCSv were 0.284 ± 0.038 (0.192–0.330, 6X-FFF beam) and 0.297 ± 0.036 (0.225–0.353, 10X-FFF beam) for the PTV-based plan, and 0.285 ± 0.050 (0.182–0.363, 6X-FFF beam) and 0.287 ± 0.054 (0.195–0.379, 10X-FFF beam) for the robust optimized plan.

## Discussion

One goal in lung VMAT SBRT is to achieve dosimetric accuracy to the tumour within limited treatment times, while keeping the surrounding dose to a minimum. We investigated the feasibility of achieving a robust optimization with a 10X-FFF beam for lung VMAT SBRT when applying a breath-holding technique and evaluated the robustness under perturbed doses shifted from the isocentre. The robust optimized plans were also compared with the PTV-based optimized plans for the ITV dose coverage, lung doses, HI, CI, MU, and delivery time. A comparison of the PTV-based optimized plans demonstrated that there were no significant differences in the ITV doses for the 6X- and 10X-FFF beams. The CIs were similar for the 6X- and 10X-FFF beams, as demonstrated in the study by Vieillevigne et al. [16]. It is more difficult to achieve a superior HI with a 10X-FFF beam than with a 6X-FFF beam. The inhomogeneity within the ITV seems to be clinically acceptable for SBRT of the lung. For the 10X-FFF beam, there was a greater increase in the dose to the lung compared to those of the 6X-FFF plans, because a 6X-FFF beam has a sharper penumbra than that of a 10X-FFF beam at a shallow depth as well as for small fields, which is presumably due to shorter secondary electron paths [24]. The MUs of both the 6X- and 10X-FFF beams were similar owing to the relatively small PTV size in our study. The delivery of the 10X-FFF beam exhibited a reduced treatment time owing to the high dose rate. For the comparison of robust optimized plans, the doses to the lung for the 10X-FFF beam were significantly higher than those for the 6X-FFF beam. As with the PTV-based optimized plans, it is more difficult to achieve a superior HI with a 10X-FFF beam than with a 6X-FFF beam. The D_99%_ dose to the ITV using the 10X-FFF beam was lower than that using the 6X-FFF beam. This difference is generally too small to be visualized on a DVH and is unlikely to be clinically significant. In addition, no recommendation for optimal value for target dose heterogeneity from planning and toxicity perspective in SBRT literature [25]. Therefore, at present HI parameter appears no significant issue in lung SBRT. Because D_95%_ at PTV was 100% and D_2%_ was ranged from 125% to 130%, dose to the ITV for both optimized plans were similar on nominal DVH and dose distribution, as shown in Figs. 1 and 2. No significant difference was found in dose to the ITV D_99%_ between PTV-based and robust optimized plans with the 6X- and 10X-FFF beams. Regarding the lung, the robust optimized plans provided a reduction in the lung involvement compared with the PTV-based optimized plans. The CIs were similar between PTV-based and robust optimized plans. This means that robust optimized plans could be created with a similar plan quality as PTV-based plans.

Patient setup errors and organ motion may lead to a dose delivery distribution that deviates from the planned dose distribution. We evaluated the plan robustness using perturbed doses shifted from the isocentre. The dose uncertainties described in this study are related to the patient motion encountered in SBRT. Dose uncertainties to the CTV using PTV-based optimized plans have been reported in the literature [10, 13]. As for robust optimized plan comparison, different doses to the lung were observed for the 6X- and 10X-FFF VMAT robust optimized plans in the perturbed dose evaluation. This is an expected result because a difference occurs even under a nominal dose distribution. Our results demonstrated that 6X-FFF beams guarantee a better HI and CI with a more sparing dose to the lung, compared with those of a 10X-FFF beam. Radiation pneumonitis is one of the major toxicities limiting the maximal radiation dose that can be safely delivered to a thoracic tumour. The reported values of V_20Gy_ < 10% and a mean dose of < 6 Gy are considered reasonable for minimising the likelihood of developing symptomatic (grade 2) pneumonitis after SBRT [4]. Our results indicate that the dose to the lung is much smaller than the aforementioned criteria. The perturbed dose evaluation reduces the dose to the ITV with no notable change in the dose to the lung compared with the nominal plans. We applied a setup error of 5 mm and obtained different D_99%_ doses to the ITV for the PTV-based and robust optimized plans using 10X-FFF beams, which were approximately 301.0 cGy and 271.6 cGy lower as compared to the nominal plan, respectively. In addition, the ITV D_99%_ dose variation for the robust optimized plan was less than that of the PTV-based optimized plan. For the comparisons of the PTV-based and robust optimized plans, the uncertainty in the ITV dose coverage was also improved by the robust optimization, particularly for a 6X-FFF beam. Our results demonstrated that 6X-FFF beams with robust optimized plans guarantee a better HI and CI with a more sparing dose to the lung compared with those of the PTV-based optimized plans. The robust optimized plans achieved more sparing doses to the lung, despite small variations in the dose to the ITV, demonstrating the advantages of robust optimized plans over PTV-based optimized plans. Some studies investigated between plan complexity and dosimetric impact for respiratory motion, reported the interplay effect was greater for more modulated plan [26, 27]. Uncertainty inter-breathhold tumor position with more complexity plan may be resulted in an interplay effect. Therefore, even if robust optimization is a useful method, increasing the plan complexity should be avoided. Our study assessed the plan complexity of robust optimization in lung cancer treatment. The average MCSv for both optimized plans was similar, because the both optimized plans were the less complicated optimization due to similar dose constraint for ring.

One potential limitation of the present study is that other OARs, such as the spinal cord, trachea, and esophagus, were not evaluated because these have previously been compared for 6X- and 10X-FFF beams for a lung SBRT in the literature [17]. In addition, although the 6X-FFF beam increases the dose to the skin, the skin dose is associated with tumours very close to the skin, beam arrangement or immobilization devices. The use of multiple beams for VMAT can reduce the dose to skin [28]. Another limitation was that robust optimization was conducted on the ITV because the CTV does not account for the breath-hold uncertainty between each breath-hold of the patient. The ITV or lung densities might affect the dose stability with robust optimization [15]. Further studies should investigate the impact of these tissue densities. It should be noted that beams with a high dose rate can deliver a substantial dose to the wrong location within a short time when applied to the treatment of a moving target. It has been demonstrated through vertebral SBRT that FFF beams with a high dose rate can cause significant dosimetric deviations even during brief intrafractional shifts [29]. In contrast, shorter treatment times can reduce the possibility of an intrafractional baseline shift or a drift in the tumour position [30]. Hanley et al. reported that a comfortable breath-holding duration for lung cancer patients is 12–16 s [31]. Thus, our data suggest that applying a robust optimization with a 10X-FFF beam for lung VMAT SBRT improves the feasibility of the breath-holding technique. It depends on the management of respiratory motion. Free breathing using abdominal compression or tracking with fiducial markers might be appropriate to use 6X-FFF. It should be carefully selected which energy is used for lung SBRT. It should also be emphasized that our study was focused on the dose uncertainty of the ITV for a robust optimization, and plan normalization was not the main objective of the present study. We did not re-normalize at ITV D_99%_ for the robust optimized plan to ensure ITV dose coverage in the worst-case scenarios of 5 mm setup errors. Robust optimized plans could provide sufficient target coverage by normalizing the ITV D_99%_ prescription dose in extreme setup error scenarios. In this study, a maximum dose variation was considered under the worst scenario for only one fraction. In addition, the same extreme error scenario is unlikely to be repeated for all fractions. Further research is required to evaluate the perturbed dose using an actual error scenario and to define the dose prescription. A comparison of large-scale multi-institutional planning of a lung SBRT demonstrated the variability in the distribution of prescription approaches, as well as between optimization strategies selected by the planner, via a multivariate analysis [32]. To compare all clinical outcomes, GTV/CTV D_50%_ and D_98%_ should be reported because a PTV prescription does not predict the dose to the GTV/CTV [12]. The dose prescription to the GTV/ITV should be determined for robust optimization in lung SBRT, while considering the dose to the lung.

## Conclusions

A 6X-FFF beam with the robust optimization was found to be preferable in the studied cases because it offers a more sparing dose to the lung. Nevertheless, owing to the available high dose rate, a 10X-FFF beam could be preferentially used for breath-holding treatment if the plan quality is acceptable. 10X-FFF beam applied under a robust optimized plan for lung SBRT cancer treatment using a breath-holding technique is a feasible method, despite a higher dose to the lung, compared to a 6X-FFF beam.

### Presentation

None.

## Conflict of interest

The authors declare that they have no conflict of interest to disclose.

## Funding

This work was supported by JSPS KAKENHI Grand Number 18 K15592.
